# Glyceroglycolipids from the solid culture of *Ophiocordyceps sinensis* strain LY34 isolated from Tibet of China

**DOI:** 10.1080/21501203.2022.2036841

**Published:** 2022-02-22

**Authors:** Baosong Chen, Jinghan Lin, Ao Xu, Dan Yu, Dorji Phurbu, Huanqin Dai, Yi Li, Hongwei Liu

**Affiliations:** a State Key Laboratory of Mycology, Institute of Microbiology, Chinese Academy of Sciences, Beijing, P. R. China; b Savaid Medicine School, University of Chinese Academy of Sciences, Beijing, P. R. China; c School of Food Science and Engineering, Yangzhou University, Yangzhou, P. R. China; d Tibet Plateau Institute of Biology, Lhasa, P. R. China

**Keywords:** *Ophiocordyceps sinensis*, digalactosyldiacylglycerol, cytotoxicity, anti-inflammatory activity, bioactive compound

## Abstract

*Ophiocordyceps sinensis* is a well-known entomogenous fungus with its fruiting bodies or cultural mycelium as food and herbal medicines in Asia. While metabolites which could responsible for its potent pharmaceutical effects has long remained to be elucidated. In this work, chemical investigation on the solid culture of *O. sinensis* strain LY34 led to the discovery of six digalactosyldiacylglycerols (DGDGS, **1–6**) including one new. The structure of compound **1** was determined based on the comprehensive spectra analysis, including NMR, MS^n^, IR, and chemical derivatisation. Bioactivity studies showed a weak cytotoxicity of compounds **1–6** against 293 T cell and medium anti-inflammatory activity of compounds **1** and **2** on Raw 264.7 cell. The discovery of DGDGs in *O. sinensis* provides new insight into the pharmacologically active substances.

## 1. Introduction

*Ophiocordyceps sinensis* (Berk.) G.H. Sung, J.M. Sung, Hywel-Jones & Spatafora (syn. *Cordyceps sinensis*), traditionally called “Dong Chong Xia Cao” in Chinese, is endemic to the alpine meadows of the Tibetan Plateau and its surrounding regions (Li et al. 2011). The fungus parasitizes larvae of ghost moths within the family Hepialidae, spreading the mycelium through the host larvae and digesting its inner organs, and then converts them into sclerotia from which the fungal fruiting body grows (Wang and Yao 2011). As a well-known Traditional Chinese Medicine, its price increased sharply in this century, the gathering of this fungus had provided a critical household income for more than one million people who lived in the production areas or nearby regions. While on the other hand, the natural resources of this species decreased due to over-harvesting and climate change (Yan et al. 2017; Hopping et al. 2018). The species has been listed as the vulnerable in the recent released “RedList of China’s Biodiversity – Macrofungi” (Yao et al. 2020) and the IUCN Red List of Threatened Species (Yang 2020), and has been included in the recent National Key Protected Wild Plants List in China under the Second-Class Category by the National Forestry and Grassland Administration and the Ministry of Agriculture and Rural Affairs (http://www.forestry.gov.cn/main/5461/20210908/162515850572900.html).

This fungal species has been traditionally used as a food and herbal medicine commonly for the treatment of kidney and lung problems for centuries (Li et al. 2002, 2015; Rakhee et al. 2021). Early studies have proven that *O. sinensis* possesses various pharmacological effects, including immunomodulating (Chiu et al. 1998; Koh et al. 2002), hypocholesterolaemic (Koh et al. 2003), antitumour (Bok et al. 1999), and antioxidant activity (Yamaguchi et al. 2000; Cho et al. 2003). Due to its particular high economic value and limited natural resources, mycelia fermentation and artificial cultivars have been developed. However, metabolites contributing to its therapeutic efficacy has not been fully elucidated from this fungus, while several constituents such as polysaccharides, mannitol, and ergosterol were reported to possess kinds of potential pharmacological activities (Leung et al. 2006).

To find pharmacologically active chemical compounds from this fungus, a systematic chemical investigation of a well-defined solid cultural medium of the *Ophiocordyceps sinensis* strain LY34 that collected from the central production area (Nagqu, Tibet) was carried out in this study. Six glyceroglycolipids were obtained and their cytotoxicity against 293 T cell lines and anti-inflammatory activity on Raw 264.7 cell lines were evaluated.

## 2. Material and method

### 2.1. General experimental procedures

UV, IR, optical rotations, and ECD spectra were obtained on a Thermo Genesys-10S UV-vis spectrophotometer, Nicolet IS5 FT-IR spectrophotometer, Anton Paar MCP 200 Automatic Polarimeter and Applied Photophysics Chirascan spectropolarimeter, respectively. NMR spectral data were recorded with a Bruker Avance-500 spectrometer in DMSO-*d*_6_ (*δ*_H_ 2.50/*δ*_C_ 39.52) or C_5_D_5_N (*δ*_H_ 8.74, 7.58 and 7.22/*δ*_C_ 150.4, 135.9 and 123.9). HSQC and HMBC experiments were optimised for 145.0 and 8.0 Hz, respectively. HRESIMS data were obtained on an Agilent Accurate-Mass-Q-TOF LC/MS 6520 instrument. HPLC analysis data were collected using Waters-2695-2998. GC-MS data were acquired by GCMS-QP2010 Ultra.

Solvents of analytical grade including methanol, dichloromethane, petroleum ether and ethyl acetate used for extraction and chromatographic separation were purchased from Beijing Chemical Works. Chromatographically pure methanol and acetonitrile used for HPLC analysis were purchased from Thermo Fisher Scientific. Thin layer chromatography (TLC) was carried out on silica gel HSGF254 plates and the spots were visualised by UV at 254 nm or sprayed with 10% H_2_SO_4_ followed by heating. Silica gel (150−250 μm, Qingdao Haiyang Chemical Co., Ltd.) were used for column chromatography (CC). HPLC separation was performed on Shimadzu LC-6AD with SPD-20A detector using a reversed phase chromatography column (C_4_, 250 × 9.4 mm, Kromasil, 5 µm) at a flow rate of 2.0 mL/min.

### 2.2. Fungal material and fermentation

Strain LY34 was isolated from inner sclerotium tissues of a fresh *Ophiocordyceps sinensis* individual collected from Nagqu, Tibet on 3 June 2018. The stock strains were maintained at 4°C on Potato Dextrose Agar (PDA) supplemented with 5% wheat bran and 0.5% peptone. Seed cultures were inoculated into 500 mL Erlenmeyer flasks containing 100 mL of wheat bran liquid culture medium (same medium as above without agar) with a 5-mm agar discs for each stock. The flasks were rotated at 18°C, 120 rpm for 20 days. Canning glass jars containing 20 g rice, 2 g oats and 40 mL fresh water were sterilised and then inoculated with 5 mL of seed culture and incubated in a biochemical incubator at a humidity of 60%, 18°C for 50 days.

### 2.3. Extraction and isolation

The solid culture was freeze-dried, and repeatedly extracted with ethyl acetate at room temperature for three times (each time, 3 L). The organic solvent was evaporated to dryness under vacuum to afford the crude extract (39 g). The crude extract was separated by silica gel column chromatography (CC) using the dichloromethane-methanol (D-M: 100:0, 20:1, 10:1, 8:1, 6:1, 4:1, 2:1, v/v) gradient elution to give 10 fractions (F1 to F10) based on TLC and HPLC analysis.

F7 (D-M, 8:1, 2.2 g) containing compounds with both hydrophilic and hydrophobic characteristic which was speculated from the large polarity in TLC and high retention in HPLC, indicated the existence of glucoside. Whereafter, F7 was subjected to Sephadex LH-20 CC, eluted with dichloromethane-methanol (1:3, v/v) to give seven sub-fractions (F7-1 to F7-7). F7-2 (80 mg) was separated by semipreparative HPLC (C4, MeOH-H_2_O, 98%, 2 mL/min) to yield compounds **1** (5.8 mg, *t*_R_ = 45.4 min) and **2** (4.6 mg, *t*_R_ = 53.2 min). And F7-4 (320 mg) was purified by HPLC (C4) using 92% MeOH in water at a flow rate of 2 mL/min to give **3** (21.1 mg, *t*_R_ = 35.1 min), **4** (8.5 mg, *t*_R_ = 42.6 min), **5** (6.3 mg, *t*_R_ = 50.4 min) and **6** (7.2 mg, *t*_R_ = 59.4 min).

Compound (**1**): colourless oil, [*α*]^25^_D_ +35 (*c* 0.1, CH_2_Cl_2_); IR (neat) *ν*_max_ 3413, 2924, 2832, 1740, 1652, 1377, 1223, 1073, 920 cm^−1^; negative HRESIMS data at *m*/*z* 1265.8513 [M+ HCOO]^−^ (calcd. for C_70_H_121_O_19_, 1265.8502) indicated the formula C_69_H_120_O_17_; ^1^H and ^13^C NMR data, see Table 1.

**Table 1.** ^1^H and ^13^C NMR data for compound **1** in pyridine-*d*_5._

Position	*δ*_C_, Carbon types	*δ*_H_ multi (*J* in Hz)	Position	*δ*_C_	*δ*_H_ multi (*J* in Hz)
Glycerol moiety			17”	19.41, CH_2_	1.36 m
1	63.76, CH_2_	4.70 m	18”	14.44, CH_3_	0.91 t (7.4)
		4.54 m	16’ or 16”‘	32.08/32.49, CH_2_	1.23 m
2	71.39, CH	5.67 m	17’ or 17”‘	23.20/23.32, CH_2_	1.23 m
3	68.41, CH_2_	4.36 m	18’ or 18”‘	14.60/14.65, CH_3_	0.87 t (7.4)
		4.04 dd (10.6, 5.3)	Others	29.30–30.60, CH_2_	1.27 m
Fatty acid ester moiety			β-d-Gal*p* moiety		
1’	173.66, C		1”“	105.91, CH	4.76 d (7.6)
1”	173.64 C		2”“	72.55, CH	4.42 m
1”‘	173.49, C		3”“	75.43, CH	4.12 dd (9.5, 3.1)
2’ or 2”	34.82/34.58, CH_2_	2.37 m	4”“	70.22, CH	4.54 m
2”‘	35.15, CH_2_	2.44 m	5”“	74.95, CH	4.18 t (6.1)
3’ or 3”	25.57/25.58, CH_2_	1.69 m	6”“	68.6, CH_2_	4.50 m
3”‘	25.83, CH_2_	1.64 m			4.36 m
8’, 15’ or 8”, 8”‘, 15”‘	27.50–28.00, CH_2_	2.00 m			
9’, 10’, 12’,13’ or 9”, 10” or 9”‘, 10”‘, 12”‘,13”‘	128.30–131.40, CH	5.51 m	α-d-Gal*p* moiety		
11’ or 11”‘	26.40/26.53, CH_2_	2.95 m	1”“‘	101.74, CH	5.51 d (3.6)
12”	131.42, CH	5.63 m	2”“‘	73.26, CH	4.56 m
13”	125.74, CH	5.57 m	3”“‘	71.39, CH	4.68 m
14”	32.84, CH_2_	2.47 m	4”“‘	71.00, CH	4.67 m
15”	73.74, CH	5.18 m	5”“‘	72.09, CH	4.55 m
16”	36.58, CH_2_	1.63 m	6”“‘	62.95, CH_2_	4.42 m

Compound (**2**): colourless oil; negative HRESIMS data at *m*/*z* 1263.8401 [M+ HCOO]^−^ (calcd. for C_70_H_119_O_19_, 1263.8347) indicated the formula C_69_H_118_O_17_; ^1^H NMR (500 MHz, DMSO-*d*_6_) *δ* 5.41 (m), 5.38–5.19 (overlap), 5.11 (m), 4.87 (m), 4.81 (p, *J* = 6.3 Hz), 4.71 (m), 4.68 (d, *J* = 3.7 Hz), 4.51 (t, *J* = 5.7 Hz), 4.48 (d, *J* = 4.9 Hz), 4.47 (d, *J* = 5.9 Hz), 4.34 (d, *J* = 4.4 Hz), 4.33 (d, *J* = 2.0 Hz), 4.31 (dd, *J* = 12.3, 3.0 Hz), 4.14 (d, *J* = 7.3 Hz), 4.12 (d, *J* = 3.8 Hz), 4.11 (d, *J* = 3.5 Hz), 3.80 (dd, *J* = 10.9, 5.4 Hz), 3.69 (m), 3.65–3.53 (overlap), 3.53–3.47 (overlap), 3.43 (dt, *J* = 11.1, 5.7 Hz), 3.28 (m), 2.73 (m), 2.24 (m), 2.01 (q, *J* = 6.9 Hz), 1.49 (m), 1.40–1.17 (overlap), 0.85 (overlap); ^13^C NMR (126 MHz, DMSO-*d*_6_) *δ* 172.51, 172.46, 172.24, 130.19, 129.84, 129.70, 129.67, 129.62, 129.62, 127.76, 127.74, 127.72, 127.70, 127.40, 124.69, 103.72, 99.57, 73.13, 72.93, 72.46, 71.28, 70.31, 69.91, 69.60, 68.85, 68.41, 67.95, 66.64, 66.42, 62.42, 60.58, 35.34, 33.77, 33.55, 33.38, 31.61, 30.91, 29.05, 28.93, 28.73, 28.70, 28.68, 28.65, 28.60, 28.58, 28.55, 28.47, 28.43, 28.39, 26.67, 26.64, 26.61, 26.58, 25.28, 25.20, 24.56, 24.44, 24.40, 22.10, 21.98, 18.12, 13.90, 13.70

Compound (**3**): colourless oil; negative HRESIMS data at *m*/*z* 985.6135 [M+ HCOO]^−^ (calcd. for C_52_H_89_O_17_, 985.6105) indicated the formula C_51_H_88_O_15_; ^1^H NMR (500 MHz, DMSO-*d*_6_) δ 5.41 (m), 5.38–5.19 (overlap), 5.10 (m), 4.88 (d, *J* = 3.4 Hz), 4.71 (m), 4.67 (d, *J* = 3.7 Hz), 4.51 (t, *J* = 5.7 Hz), 4.48 (d, *J* = 4.9 Hz), 4.47 (d, *J* = 5.9 Hz), 4.35 (d, *J* = 6.7 Hz), 4.34 (d, *J* = 3.7 Hz), 4.31 (dd, *J* = 12.1, 3.0 Hz), 4.14 (d, *J* = 7.3 Hz), 4.12 (d, *J* = 3.8 Hz), 4.11 (d, *J* = 3.5 Hz), 3.80 (dd, *J* = 10.9, 5.4 Hz), 3.69 (m), 3.63–3.53 (overlap), 3.53–3.47 (overlap), 3.43 (dt, *J* = 11.0, 5.7 Hz), 3.28 (overlap), 2.73 (t, *J* = 6.5 Hz), 2.27 (m), 2.01 (q, *J* = 6.9 Hz), 1.50 (m), 1.40–1.17 (overlap), 0.85 (t, *J* = 6.9 Hz); ^13^C NMR (126 MHz, DMSO-*d*_6_) δ 172.56, 172.28, 129.72, 129.65, 127.76, 127.72, 103.72, 99.55, 73.13, 72.94, 71.28, 70.31, 69.92, 69.60, 68.86, 68.41, 67.98, 66.65, 66.44, 65.03, 62.41, 60.59, 33.56, 33.38, 30.91, 29.04, 28.73, 28.64, 28.61, 28.57, 28.55, 28.44, 28.41, 26.64, 26.61, 25.21, 24.44, 24.41, 21.98, 13.92.

Compound (**4**): colourless oil; negative HRESIMS data at *m*/*z* 961.6187 [M+ HCOO]^−^ (calcd. for C_50_H_89_O_17_, 961.6105) indicated the formula C_49_H_88_O_15_; ^1^H NMR (500 MHz, DMSO-*d*_6_) δ 5.31 (overlap), 5.10 (m), 4.88 (m), 4.71 (m), 4.67 (d, *J* = 3.6 Hz), 4.51 (t, *J* = 5.7 Hz), 4.48 (d, *J* = 4.9 Hz), 4.47 (d, *J* = 5.9 Hz), 4.35 (d, *J* = 6.7 Hz), 4.34 (d, *J* = 3.7 Hz), 4.31 (dd, *J* = 12.1, 3.0 Hz), 4.14 (d, *J* = 7.3 Hz), 4.12 (d, *J* = 3.8 Hz), 4.11 (d, *J* = 3.5 Hz), 3.80 (dd, *J* = 10.9, 5.4 Hz), 3.69 (m), 3.64–3.53 (overlap), 3.53–3.47 (overlap), 3.43 (m), 3.28 (m), 2.72 (t, *J* = 6.6 Hz), 2.26 (m), 2.01 (q, *J* = 6.9 Hz), 1.49 (t, *J* = 7.1 Hz), 1.37–1.19 (overlap), 0.85 (overlap); ^13^C NMR (126 MHz, DMSO-*d*_6_) δ 172.57, 172.27, 129.71, 129.64, 127.75, 127.71, 103.73, 99.56, 73.14, 72.95, 71.29, 70.33, 69.93, 69.61, 68.87, 68.42, 67.99, 66.66, 66.45, 62.43, 60.60, 33.58, 33.43, 31.33, 30.93, 29.08, 29.05, 28.96, 28.78, 28.75, 28.68, 28.61, 28.47, 28.45, 26.67, 26.63, 25.22, 24.46, 24.45, 22.12, 22.00, 13.95, 13.91.

Compound (**5**): colourless oil; negative HRESIMS data at *m*/*z* 987.6277 [M+ HCOO]^−^ (calcd. for C_52_H_91_O_17_, 987.6256) indicated the formula C_51_H_90_O_15_; ^1^H NMR (500 MHz, DMSO-*d*_6_) δ 5.37–5.25 (overlap), 5.11 (m), 4.88 (m), 4.71 (m), 4.68 (d, *J* = 3.6 Hz), 4.51 (t, *J* = 5.7 Hz), 4.48 (d, *J* = 4.9 Hz), 4.47 (d, *J* = 5.9 Hz), 4.35 (d, *J* = 6.7 Hz), 4.34 (d, *J* = 3.7 Hz), 4.31 (dd, *J* = 12.1, 3.0 Hz), 4.14 (d, *J* = 7.3 Hz), 4.12 (d, *J* = 3.8 Hz), 4.11 (d, *J* = 3.5 Hz), 3.80 (dd, *J* = 10.9, 5.4 Hz), 3.69 (m), 3.64–3.53 (overlap), 3.53–3.47 (overlap), 3.43 (m), 3.28 (m), 2.72 (t, *J* = 6.6 Hz), 2.26 (m), 2.01 (q, *J* = 6.7 Hz), 1.97 (q, *J* = 6.4, 5.9 Hz), 1.49 (t, *J* = 7.1 Hz), 1.37–1.19 (overlap), 0.85 (overlap); ^13^C NMR (126 MHz, DMSO-*d*_6_) δ 172.53, 172.26, 129.70, 129.63, 129.61, 129.55, 127.75, 127.71, 103.73, 99.57, 73.14, 72.94, 71.29, 70.32, 69.91, 69.61, 68.86, 68.42, 67.97, 66.65, 66.43, 62.43, 60.59, 33.57, 33.40, 31.30, 30.93, 29.14, 29.12, 29.06, 28.86, 28.75, 28.71, 28.66, 28.64, 28.62, 28.59, 28.57, 28.54, 28.46, 28.43, 26.65, 26.62, 26.58, 25.21, 24.45, 24.42, 22.11, 21.99, 13.93, 13.91.

Compound (**6**): colourless oil; negative HRESIMS data at *m*/*z* 963.6275 [M+ HCOO]^−^ (calcd. for C_50_H_91_O_17_, 963.6256) indicated the formula C_49_H_90_O_15_; ^1^H NMR (500 MHz, DMSO-*d*_6_) δ 5.31 (overlap), 5.10 (m), 4.88 (d, *J* = 3.4 Hz), 4.71 (m), 4.67 (d, *J* = 3.6 Hz), 4.51 (t, *J* = 5.7 Hz), 4.48 (d, *J* = 4.9 Hz), 4.47 (d, *J* = 5.9 Hz), 4.35 (d, *J* = 6.7 Hz), 4.34 (d, *J* = 3.7 Hz), 4.31 (dd, *J* = 12.1, 3.0 Hz), 4.14 (d, *J* = 7.3 Hz), 4.12 (d, *J* = 3.8 Hz), 4.11 (d, *J* = 3.5 Hz), 3.80 (dd, *J* = 10.9, 5.4 Hz), 3.68 (m), 3.63–3.53 (overlap), 3.53–3.47 (overlap), 3.43 (ddd, *J* = 11.1, 6.4, 5.1 Hz), 3.28 (m), 2.26 (m), 1.97 (q, *J* = 6.4 Hz), 1.49 (m), 1.23 (overlap), 0.85 (t, *J* = 6.8 Hz); ^13^C NMR (126 MHz, DMSO-*d*_6_) δ 172.57, 172.28, 129.62, 129.56, 103.72, 99.55, 73.13, 72.95, 71.28, 70.32, 69.92, 69.60, 68.87, 68.41, 67.99, 66.66, 66.45, 65.03, 62.42, 60.59, 33.58, 33.42, 31.30, 29.15, 29.11, 29.07, 29.03, 28.94, 28.86, 28.77, 28.73, 28.71, 28.66, 28.61, 28.57, 28.46, 28.43, 26.63, 26.58, 24.46, 24.44, 22.11, 13.95.

### 2.4. Alkaline hydrolysis reaction and GC-MS analysis

Each compound (1.0 mg) was dissolved in CH_2_Cl_2_ and hydrolysed with 2 M NaOH/MeOH (2 mL) for 3 h at room temperature. The resulting mixture was then treated with 10 mL of HCl/MeOH under stirring for overnight. The mixture containing methyl ester of fatty acids was extracted with CH_2_Cl_2_ for two times (10 mL × 2). The organic layer was evaporated to dryness under vacuum and then dissolved in 1 mL chromatographically pure CH_2_Cl_2_ followed by GC-MS analysis. The samples were analysed in split injector mode by using a fused silica capillary column Rtx-5 MS (crosslinked 5% diphenyl dimethyl polysiloxane, 30 m × 0.25 mm ID × 0.25 μm) with helium (1 mL/min) as carrier. Oven temperatures were programmed from 50 to 325°C at a slope of 10°C per minute and then hold for 15 minutes. The MS was operated in EI mode (70 eV) scanning from 40 to 500 amu. The retention time of methyl oleate and methyl linoleate was 18.80 and 18.83 min, respectively (Chen et al. 2020a).

### 2.5. Acid hydrolysis and determination of monosaccharides

Based on the literature (Åman et al. 1981), each glycoside (2.0 mg) was hydrolysed with 2 M HCl (2 mL) for 3 h at 90℃. After three times extraction with CHCl_3_ (3 × 5 mL), the aqueous layer was evaporated to dryness under vacuum. Take up the residue into two quantities using for PMP (1-phenyl-3-methyl-5-pyrazolone) derivatisation and absolute configuration determination.

The procedure employed for the derivatisation of monosaccharides was carried out according to the method of previous report with some modifications (Fu and O’Neill 1995). The hydrolysed residue was dissolved in 200 μL 0.3 mol/L NaOH, then 0.5 M methanol solution (100 μL) of PMP was added and mixed. The mixture was allowed to react for 20 min at 70°C, then cooled to ambient temperature and was neutralised with 0.3 mol/L HCl. Water and chloroform (1.0 mL each) were added and shaken vigorously. The chloroform layer was discarded, and the extraction process was repeated three times. Then, the water layer was centrifugated at 8,000 rpm for 10 min, which was collected for analysis directly or stored at −20°C for later HPLC analysis. The analysis of PMP derivatives was conducted on Waters-2695-2998 HPLC system and Eclipse Plus C18 column (4.6 × 250 mm, 5 μm, Agilent). The operating parameters of HPLC were as follows: column oven temperature, 40°C; mobile phase, a mixture of phosphate buffered saline (PBS, 0.1 M, pH6.7) and acetonitrile at a ratio of 83:17 (v/v); flow rate, 1.0 mL/min; detector wavelength, 245 nm; injection volume, 10 μL. Standard monosaccharides were derived and analysed using the same method as above.

To confirm the absolute configuration, dried residue was dissolved in pyridine (0.5 mL, Sigma) containing l-cysteine methyl ester (1.0 mg, Sigma) and heated at 60℃ for 1 h. A solution of *O*-tolylisothiocyanate (5.0 μL, Sigma) was added to the mixture and heated at 60 ℃ for another 1 h (Wang et al. 2021). The reaction mixture was analysed by Waters-2695-2998 HPLC system and Eclipse Plus C18 column (4.6 × 250 mm, 5 μm, Agilent) to identify the derivatives of constituent monosaccharides by comparison of their retention time with those of authentic samples with mobile phase MeCN–H_2_O–1‰ formic acid (20:80, v/v), detection wavelength 254  nm, flow rate 1 mL/min and column temperature 35℃.

### 2.6. Cytotoxicity

Cell lines 293 T (human embryonic kidney) were purchased from National Infrastructure of Cell Line Resource. Cytotoxicity assay was performed according to the method previously reported (Chen et al. 2019). The inhibition rate was calculated with the following formula: Inhibitory rate (%) = [1−(OD _treated_/OD _control_)] × 100% and IC_50_ was calculated by plotting inhibitory rate vs. test concentrations.

### 2.7. Anti-inflammatory activity

The anti-inflammatory activities of compounds were evaluated using LPS-induced RAW 264.7 cells as previously reported (Chen et al. 2020b). Nitric Oxide (NO) production in each well was assessed by measuring the accumulation of nitrite in the culture medium using Griess reagent.

## 3. Result and discussion

**Figure 1.**
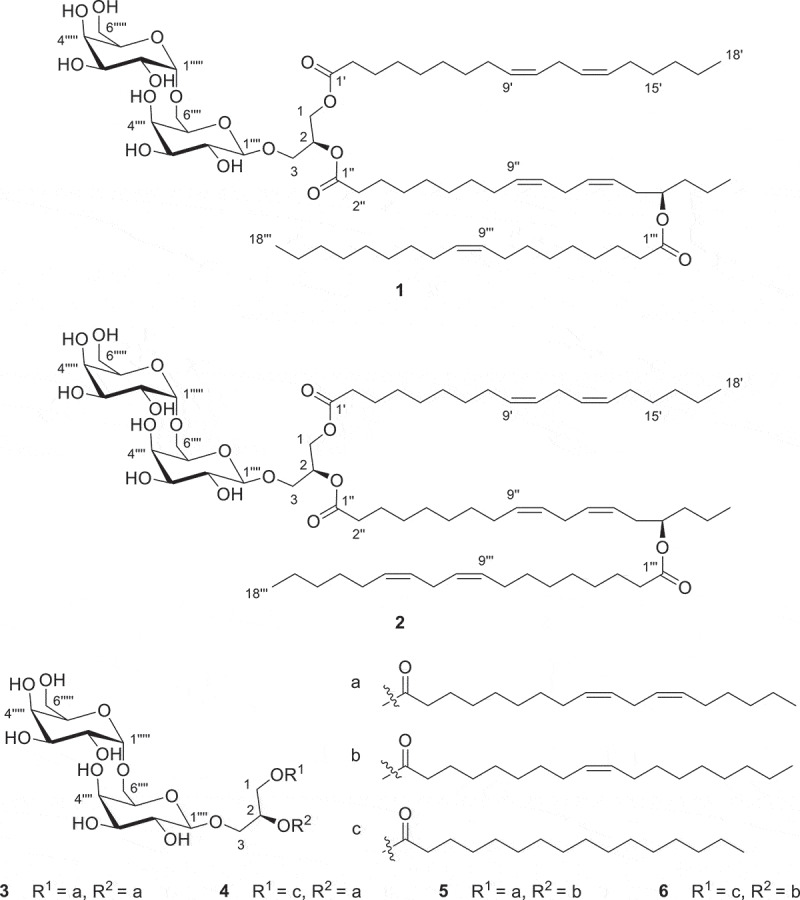
Structures of compounds **1**–**6.**

The solid culture of *O. sinensis* strain LY34 extracted with ethyl acetate gave a brown crude extract. The chromatographic separation of the extract using silica gel, Sephadex LH-20 chromatographic column and semi-preparative HPLC lead to the separation of six glyceroglycolipids (**1–6**) including one new glyceroglycolipids (**1**) (Figure 1). The structures of five known compounds were confirmed as *sn*-1-[(9′*Z*,12′*Z*)-octadecadienoyl]-2-{(15′′*R*)-[(9′′′*Z*,12′′′*Z*)-octadecadienoyloxy]-(9′′*Z*,12′′*Z*)-octadecadienoyl}-3-(α-d-galactopyranosyl-1′′′′′-6′′′′-β-d-galactopyranosyl)-glycerol (**2**) (Hamberg et al. 1998), *sn*-1-[(9′*Z*,12′*Z*)-octadecadienoyl]-2-[(9′′*Z*,12′′*Z*)-octadecadienoyloxy]-3-(α-d-galactopyranosyl-1′′′′-6′′′-β-d-galactopyranosyl)-glycerol (**3**) (Tarawneh et al. 2013), *sn*-1-[(9′*Z*,12′*Z*)-octadecadienoyl]-2-palmitoyloxy-3-(α-d-galactopyranosyl-1′′′′-6′′′-β-d-galactopyranosyl)-glycerol (**4**) (Napolitano et al. 2007; Gao et al. 2008), *sn*-1-[(9′*Z*,12′*Z*)-octadecadienoyl]-2-{(15′′*R*)-[(9′′′*Z*,12′′′*Z*)-octadecadienoyloxy]-(9′′*Z*,12′′*Z*)-octadecadienoyl}-3-(α-d-galactopyranosyl-1′′′′′-6′′′′-β-d-galactopyranosyl)-glycerol (**5**) (Falsone et al. 1995), *sn*-1-palmitoyloxy-2-[(9′′*Z*)-octadecadienoyl]-3-(α-d-galactopyranosyl-1′′′′-6′′′-β-d-galactopyranosyl)-glycerol (**6**) (Gao et al. 2008) by comparing their spectroscopic data with the literature data. The structure of new compound was elucidated by extensive spectroscopic analysis.

**Figure 2.**
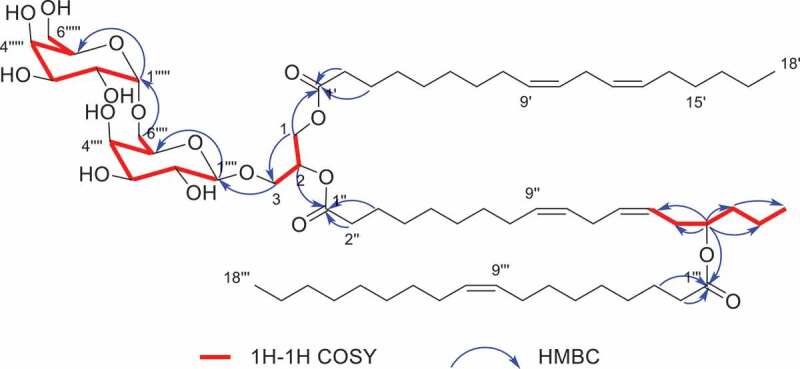
Key ^1^H-^1^H COSY and HMBC correlations of compound **1.**

Compound **1** was obtained as colourless oil. Based on the negative HRESIMS data at *m*/*z* 1265.8513 [M+ HCOO]^−^ (calcd. for C_70_H_121_O_19_, 1265.8502), the molecular formula of **1** was determined to be C_69_H_120_O_17_. The ^1^H and ^13^C NMR spectrum of **1** (Table 1) exhibited representative sugars signals and lipids signals. The proton signals from *δ*_H_ 3.00 to 5.00 together with carbon signals from *δ*_C_ 60.0 to 110.0, especially two carbon signals at *δ*_C_ 101.74 and 105.91, revealed the existence of two sugars. The ^1^H-^1^H COSY correlations (Figure 2) H-1”“-H-2”“-H-3”“ and H_2_-6”“-H-5”“ and HMBC correlations (Figure 2) from the H-1”“ to C-2”“, C-3”“ and C-5”“, from H-2”“ to C-1”“ and C-3”“, from H-3”“ to C-2”“ and C-4”“, from H-4”“ to C-3”“ and C-5”“, and from H-5”“ to C-4”“, C-6”“ and C-1”“, assigned the six-carbon pyranose. Another six-carbon pyranose was constructed by analyzing the correlations as above. The HMBC correlations from H_2_-6”“ to C-1”“‘ indicated two sugars connected together via 1,6-O-glycosidic bond. And three ester carbonyl carbons at *δ*_C_ 173.66, 173.64 and 173.49, three overlapped triplet methyls at *δ*_H_ 0.85–0.92 and *δ*_C_ 14.44, 14.60 and 14.65, and the overlapped signals at *δ*_H_ 1.26 and from *δ*_C_ 29.30 to 30.60 indicated three fatty acid moieties in the structure. Analyzing ^1^H-^1^H COSY correlations H-2 with H_2_-1 and H_2_-3 and HMBC correlations from the H_2_-1 to C-2 and C-3, the left carbon signals in the region of *δ*_C_ 60.0 to 80 were attributed to the glycerol moiety. The HMBC correlations from H_2_-1 to C-1’, from H-2 to C-1”, from H_2_-3 to C-1”“ proves two fatty acids and one sugar link to the C-1, C-2 and C-3 by ester bond and glycosidic bond. The HMBC correlations from H-15” to C-1”‘, from H-15” to C-13”, C-14”, C-16” and C-17”, and from H_3_-18” to C-16” and C-17” indicated the third fatty acid linking to aliphatic chain of another fatty acid moiety.

**Figure 3.**
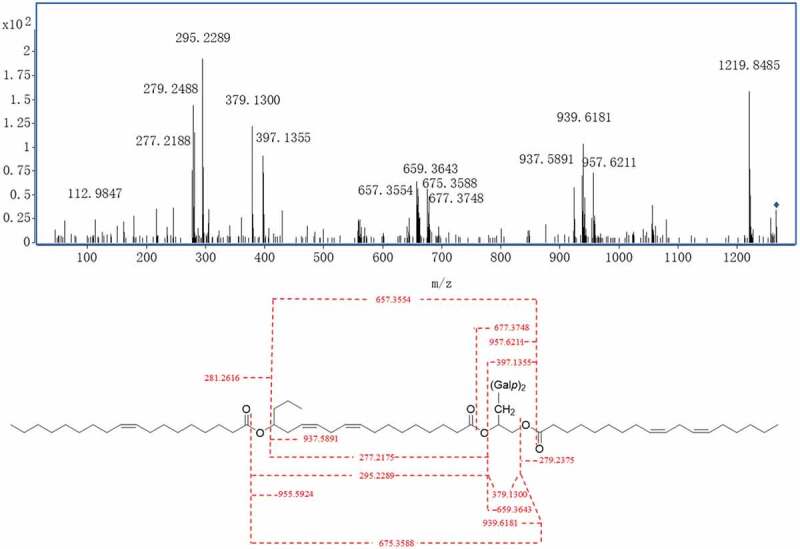
MS/MS spectrum and the plausible formation of MS/MS molecular ion fragments of compound **1.**

**Figure 4.**
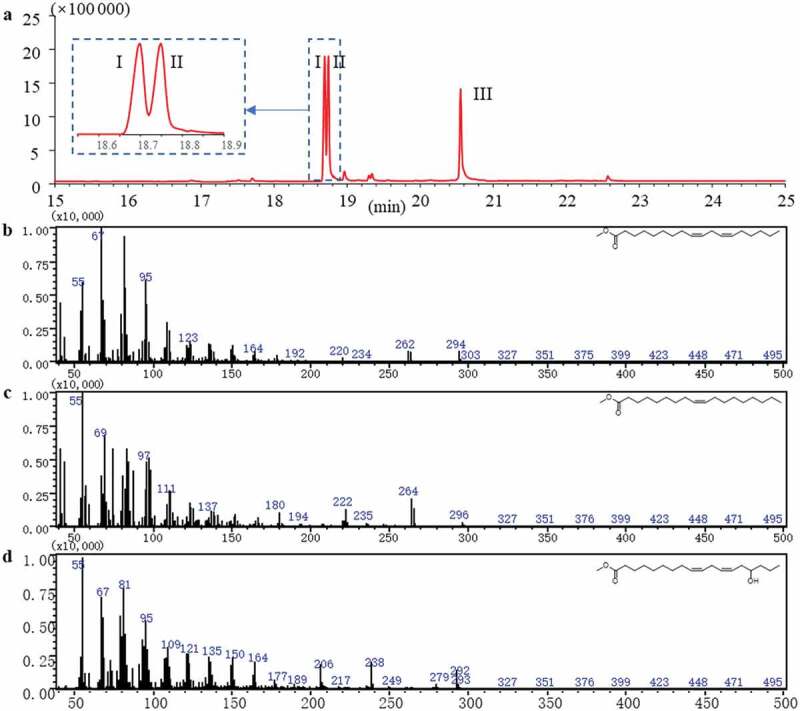
GCMS analysis of hydrolysate of compound **1. a**, GC chromatogram of compound **1; b-d**, mass spectrogram of peak I–III in **a**, which was assigned as methyl linoleate, methyl oleate and 15-OH methyl linoleate by the database (NIST-14) searching, with the similarity of 96%, 97%, and 97%, separately.

To further confirm the structure of fatty acid moieties, its negative HRESITOF-MS/MS data were obtained. In the negative ion mode MS/MS spectrum (Figure 3), compound **1** showed a precursor ion [M-H]^−^ at *m*/*z* 1219.8485 as well as fragment ions at *m*/*z* 279.2488, 281.2616 and 295.2289, supporting the existence of fatty acid 18:2 (C_18_H_32_O_2_), 18:1 (C_18_H_34_O_2_), and the hydroxylated fatty acid 18:2 (C_18_H_32_O_3_). In addition, a fragment ion [M-H-C_18_H_32_O_2_]^−^ at *m*/*z* 939.6181 due to the loss of a molecule of fatty acid 18:2, a fragment ion [M-H-C_18_H_34_O_2_]^−^ at *m*/*z* 937.5891 for the loss of fatty acid 18:1, and a fragment ion [M-H-C_18_H_34_O_2_-C_18_H_32_O_2_]^−^ at *m*/*z* 657.3554 due to the loss of one molecule of fatty acid 18:1 and one molecule of fatty acid 18:2, and a fragment ion [M-H-C_18_H_34_O_2_-C_18_H_30_O_2_]^−^at *m*/*z* 659.3643 due to the loss of one molecule of fatty acid 18:1 and one molecule of the dehydration derivative of fatty acid 18:2 were observed. All above data proved the fatty acids in compound **1** was included the fatty acid 18:2, 18:1, and the hydroxylated fatty acid 18:2. The plausible fragments formation mechanism is shown in Figure 3. Then, compound **1** was hydrolysed with alkaline followed by methyl esterification. The three fatty acid moieties in **1** were determined to be the linoleic acid, oleic acid, and 15-OH linoleic acid by comparison of their retention time and MS spectrum with those of standards by GC-MS analysis (Figure 4).

**Figure 5.**
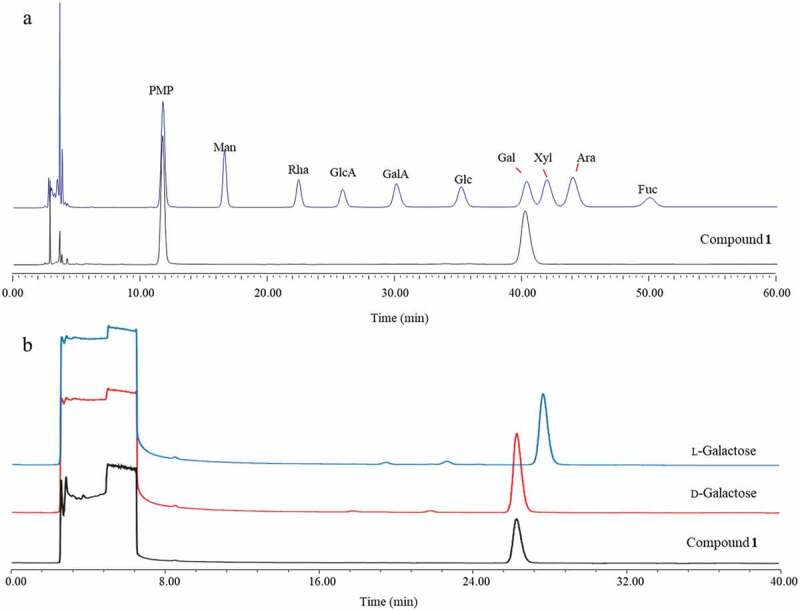
HPLC chromatogram for the PMP derivatisation (a) and absolute configuration (b) of monosaccharides in compound **1**. PMP, Man, Rha, GlcA, GalA, Glc, Gal, Xyl, Ara and Fuc were the abbreviation of 1-phenyl-3-methyl-5-pyrazolone, mannose, rhamnose, glucuronic acid, galacturonic acid, glucose, galactose, xylose, arabinose, and fucose.

To determine the kind of monosaccharides, compound **1** was hydrolysis by HCl and derived by PMP (1-phenyl-3-methyl-5-pyrazolone). Compared with the standard compounds using reversed phase HPLC, the same retention time of derivative with galactose-PMP derivative (Figure 5(a)) indicated that both of the two monosaccharides were galactoses. Whereafter, the acid hydrolysis product of compound **1**, l-galactose and d-galactose were reacted with l-cysteine methyl ester and *O*-tolylisothiocyanate. The results of HPLC showed the two monosaccharides in **1** were both d-galactoses (Figure 5(b)). The chemical shifts of C-1”“ (*δ*_C_ 105.91) and C-1”“‘ (*δ*_C_ 101.74) and coupling constants of H-1”“ [*δ*_H_ 4.76 (d, *J* = 7.6 Hz)] and H-1”“‘ [*δ*_H_ 5.51 (d, *J* = 3.6 Hz)] showed that the galactose ring proximal to the glycerol moiety is a β-anomer and that the terminal galactose ring is an α-anomer.

The absolute configuration of C-15”‘ in fatty acid chain can not be determined based on currently available data. Taking the biosynthetic relationship between **1** and **2** into consideration (Hamberg et al. 1998), the structure of **1** was deduced to be *sn*-1-[(9′*Z*,12′*Z*)-octadecadienoyl]-2-{(15′′*R*)-[(9′′′*Z*)-oleoyl]-(9′′*Z*,12′′*Z*)-octadecadienoyl}-3-(α-d-galactopyranosyl-1′′′′′-6′′′′-β-d-galactopyranosyl)-glycerol.

Digalactosyldiacylglycerols (DGDGs) play important roles in life process as the key constituent of the cell membranes. Several galactolipids have shown anti-tumour activity and anti-inflammatory activity (Syrov et al. 1991; Peterson et al. 2002; Chen et al. 2004; Zhang et al. 2012; Kolar et al. 2019). In our research, the isolated compounds were tested for cytotoxicity on cell lines 293 T. All compounds showed weak cytotoxicity against 293 T cell lines within 100 μM, with the inhibition of 23.2%, 26.3%, 21.2%, 28.9%, 33.2%, and 27.5% at 100 μM, respectively. To assay the anti-inflammation potential, the NO production in LPS-induced RAW 264.7 mouse macrophages treated with **1**–**6** were measured. At 10 μM, only compounds **1** and **2** exhibited medium inhibitory activity, and the inhibition ratio were 52.3% and 70.6%. These results indicated the double bonds were necessary for the anti-inflammatory activity of DGDGs.

In our study, one new digalactosyldiacylglycerol (**1**) and five known analogues were isolated from the mycelia of *Ophiocordyceps sinensis* Strain LY34. The structure of compound **1** was elucidated based on the comprehensive spectra analysis, including NMR, MS^n^, and IR, and chemical derivatisation. Bioactivity studies showed a weak cytotoxicity of compounds **1–6** against 293 T cell and medium anti-inflammatory activity of compounds **1** and **2** on Raw 264.7 cell. The discovery of DGDGs in *O. sinensis* provides new insight into the pharmacological substances.

## Supplementary Material

Supplemental Material
