# Supplementary material for: Glyceroglycolipids from the solid culture of Ophiocordyceps sinensis strain LY34 isolated from Tibet of China
Source: Mycology. 2022 Feb 22;13(3):185–94. doi: 10.1080/21501203.2022.2036841 (PMC9354630; doi:10.1080/21501203.2022.2036841)
Supplement: Supplemental Material [file TMYC_A_2036841_SM3577.docx]

**Supplemental information**

**Glyceroglycolipids from** **the solid culture of *Ophiocordyceps sinensis* strain LY34** **isolated from Tibet of China**

Baosong Chen ^a, #^, Jinghan Lin ^a, b, #^, Ao Xu ^c^, Dan Yu ^c^, Dorji Phurbu ^d^, Huanqin, Dai ^a, b^, Yi Li ^c, *^, Hongwei Liu ^a, b, *^

^a^ *State Key Laboratory of Mycology, Institute of Microbiology, Chinese Academy of Sciences. No.1 Beichenxi Road, Chaoyang District, Beijing, 100101, P. R. China.*

^b^ *Savaid Medicine School, University of Chinese Academy of Sciences, Beijing, P. R. China.*

^c^ *School of Food Science and Engineering, Yangzhou University, Yangzhou, Jiangsu 225127, P. R. China.*

^d^ *Tibet Plateau Institute of Biology, Lhasa 850000, P. R. China.*

# These authors contributed equally to this work.


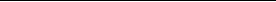


* Corresponding Author

Prof. Dr. Hongwei Liu, State Key Laboratory of Mycology, Institute of Microbiology, Chinese Academy of Sciences, No.1 Beichenxi Road, Beijing 100101, P. R. China, E-mail: liuhw@im.ac.cn; Dr. Yi Li, School of Food Science and Engineering, Yangzhou University, Yangzhou, Jiangsu 225127, P. R. China, Email: [liyi062@yzu.edu.cn](mailto:liyi062@yzu.edu.cn)

**Table of contents**

[Figure S1 ^1^H NMR spectrum of compound 1 in Pyridine-*d*_6_ (500 MHz) 3](#_Toc88550836)

[Figure S2 ^13^C NMR spectrum of compound 1 in Pyridine-*d*_6_ (125 MHz) 4](#_Toc88550837)

[Figure S3 HSQC spectrum of compound 1 in Pyridine-*d*_6_ 5](#_Toc88550838)

[Figure S4 ^1^H-^1^H COSY spectrum of compound 1 in Pyridine-*d*_6_ 6](#_Toc88550839)

[Figure S5 HMBC spectrum of compound 1 in Pyridine-*d*_6_ 7](#_Toc88550840)

**Figure S1** ^1^H NMR spectrum of compound **1** in Pyridine-*d*_6_ (500 MHz)


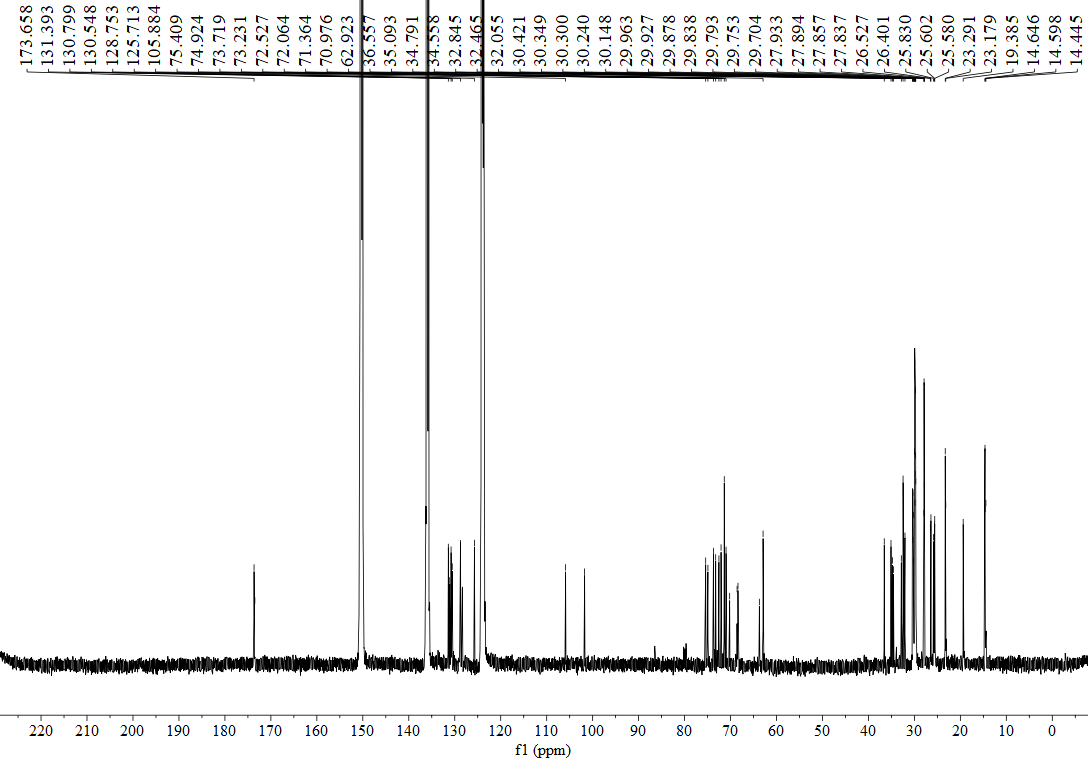


**Figure S2** ^13^C NMR spectrum of compound **1** in Pyridine-*d*_6_ (125 MHz)

**Figure S3** HSQC spectrum of compound **1** in Pyridine-*d*_6_

**Figure S4** ^1^H-^1^H COSY spectrum of compound **1** in Pyridine-*d*_6_

**Figure S5** HMBC spectrum of compound **1** in Pyridine-*d*_6_
